# Multi-Objective Optimization of Sugarcane Milling System Operations Based on a Deep Data-Driven Model

**DOI:** 10.3390/foods11233845

**Published:** 2022-11-28

**Authors:** Zhengyuan Li, Jie Chen, Yanmei Meng, Jihong Zhu, Jiqin Li, Yue Zhang, Chengfeng Li

**Affiliations:** 1 College of Mechanical Engineering, Guangxi University, Nanning 530004, China; 2 Department of Precision Instrument, Tsinghua University, Beijing 100083, China

**Keywords:** sugarcane milling system, synergetic theory, deep kernel extreme learning machine, multi-objective chicken swarm optimization, deep deterministic policy gradient

## Abstract

The extraction of sugarcane juice is the first step of sugar production. The optimal values of process indicators and the set values of operating parameters in this process are still determined by workers’ experience, preventing adaptive adjustment of the production process. To address this issue, a multi-objective optimization framework based on a deep data-driven model is proposed to optimize the operation of sugarcane milling systems. First, the sugarcane milling process is abstracted as the interaction of material flow, energy flow, and information flow (MF–EF–IF) by introducing synergetic theory, and each flow’s order parameters and state parameters are obtained. Subsequently, the state parameters of the subsystems are taken as inputs, and the order parameters—including the grinding capacity, electric consumption per ton of sugarcane, and sucrose extraction—are produced as outputs. A collaborative **optimization** model of the MF–EF–IF of the milling system is established by using a deep kernel extreme learning machine (DK-ELM). The established milling system model is applied for an improved multi-objective chicken swarm optimization (IMOCSO) algorithm to obtain the optimal values of the order parameters. Finally, the milling process is described as a Markov decision process (MDP) with the optimal values of the order parameters as the control objectives, and an improved deep deterministic policy gradient (DDPG) algorithm is employed to achieve the adaptive optimization of the operating parameters under different working conditions of the milling system. Computational experiments indicate that enhanced performance is achieved, with an increase of 3.2 t per hour in grinding capacity, a reduction of 660 W per ton in sugarcane electric consumption, and an increase of 0.03% in the sucrose extraction.

## 1. Introduction

The production of cane sugar involves sugarcane milling, juice clarification, juice evaporation, sugar crystallization, sugar paste separation, and dry product packaging. Sugarcane milling is the first step in the production of cane sugar, mainly involving heavy load equipment, the electric consumption of which accounts for more than 50% of the whole sugar production process [1,2]. Clean production is currently advocated to minimize waste and emissions while maximizing the production of manufactured products [3,4]. Whether production indicators such as the grinding capacity, sucrose extraction, and electric consumption meet the standards will affect sugar production’s smooth operation and economic benefits. The output of the manufacturing system mainly depends on the corresponding conditions of input [5]. The appropriate setting of the operating parameters in sugarcane milling is a necessary premise to ensure that the production index fluctuates around the optimal value. Therefore, it is particularly important to study the optimization of the operational parameters in the sugarcane milling process and provide guidance for improving the energy costs, quality, and yield of the sugarcane milling system.

The optimization of the operating parameters in the production process includes two key steps: the establishment of the model, and the selection of the optimization method(s). Model building is used to regress the relationship between the operating parameters and process indices, mainly including mechanistic modeling and data-driven modeling. Then, the optimal process indices and the corresponding operating parameters are obtained by using reasonable optimization algorithms based on the established model. 

Many studies have sought to improve the performance of sugarcane milling systems by improving the milling mechanism. Adam et al. simulated the pressing of sugarcane using finite factors and studied the effect of sugarcane thickness on the extraction of cane juice, without considering the dynamic effects [6]. Duan et al. proposed a two-step method to analyze the relevant parameters of the pressing process and determine the factors that have a significant influence on the pressing process [7]. Qiu et al. proposed an arbitrary Lagrangian–Euler algorithm to simulate the dynamic process of sugarcane milling and study the corresponding changes in the physical quantities [8]. Duan et al. modeled the evolution of the properties of the sugarcane milling process using the three-dimensional simulation method of the modified Drucker–Prager cap model and concluded that the compression ratio is the most important parameter. This method might provide a more accurate prediction for the optimization of these important parameters during the milling process of sugarcane [9]. However, the sugarcane milling system consists of not only the pressing process, but also the processes of crushing, tearing, and seepage [7,10]. Hence, a mechanistic model of the process is difficult to establish. With the development of big data in the industry, data-driven modeling has become a new approach to model complex production processes that do not depend on mathematical expressions. Many studies have sought to apply such an approach in the sugar industry. Lin et al. used improved binary heuristic dynamic planning to build an Elman network model and predict sucrose juice’s neutral and transparent pH values [11]. Song et al. used the principal component analysis (PCA) method to process the production data and developed a generalized dynamic fuzzy neural network to predict the color value and acidity of the sucrose carbonation clarification process [12]. Meng et al. proposed a data-driven model based on a kernel extreme learning machine (KELM) to predict the juice’s gravity purity and the clear juice’s color value [13]. Georgieva et al. took mother-liquid oversaturation and other independent parameters as inputs, and chose crystal nucleation, growth, and aggregation as outputs, in order to establish an offline prediction model [14]. Meng et al. predicted the grain content and crystal distribution in the crystallization process based on a mechanistic, data-driven model [15].

In addition to modelling milling systems, the optimization of milling system operations considering multiple objectives should also be investigated. At present, the combination of a data-driven prediction model with an intelligent optimization algorithm is the most popular framework for studying methods for the optimization of operational parameters in process industries [16], and many scholars have proposed different multi-objective optimization models. Saleh et al. proposed a machine learning (ML) model optimization method for building energy loads for forecasting both heating and cooling loads, and they used a MOO-based multi-objective optimization with an evolutionary algorithm to search the space of possible parameters [17]. Wu et al. proposed a deep-learning-based data-driven genetic algorithm and Technique for Order Preference by Similarity to Ideal Solution (TOPSIS) for the multi-objective optimization of machining process parameters and searching for final solutions, which improved the environmental impact and production efficiency of the machining process effectively [18]. Tang et al. combined a deep belief network (DBN) and Jaya algorithm to model and optimize a combustion system; the experimental results showed that both combustion efficiency and NOx emissions were improved by using the optimal control settings of the combustion system [19]. Hao et al. used coal consumption and free calcium oxide content as optimization targets in cement calcination and proposed a time-domain rolling multi-objective Jaya algorithm (TDRM-Jaya) to optimize the dynamic crushing process [20]. Tikadar et al. established three different multi-objective optimization models based on the process safety, environment, economy, and modified an industrial gas desulfurization plant by adjusting the operating parameters [21]. Nouiri et al. set up a flexible workshop-scheduling multitarget optimization model with the processing time, energy cost, and machine completion time of sand-casting as optimization targets [22]. However, traditional heuristic algorithms do not perform well in the face of the large number of complex parameter variables and high-dimensional decision space in the process industry [23]. In contrast, deep reinforcement learning (DRL) has shown better optimization performance than heuristic algorithms in many fields [24]. He et al. proposed a decision-making optimization system for the textile chemical manufacturing process based on random forests and deep reinforcement learning, and achieved parameter optimization for the process under multi-criteria conditions [25]. Zhou et al. proposed a mathematical model of thermochemical production in heavy oil reservoirs; the deep reinforcement learning algorithm was used in the model to predict dynamic production parameters and determine the combination of parameters that maximized oil recovery [26]. Cheng et al. developed an optimization system for coal-fired boilers based on deep reinforcement learning to determine the optimal boiler operating parameters, with reducing emissions and improving fuel utilization as the objective functions [27]. However, there are few studies on the modeling and multi-objective optimization of sugarcane milling systems in the literature. Therefore, in order to fill this research gap, a multi-objective optimization framework based on a deep data-driven model is proposed in this paper. 

To address the adaptive optimization of operating parameters in the sugarcane milling system, the milling system was abstracted as the interaction of material, energy, and information flow (MF–EF–IF). Using the production targets as the order parameters of flows, we combined the mutual information and wrapper method based on hybrid chicken swarm optimization to obtain the features of each flow subsystem. Next, the deep kernel extreme learning machine (DK-ELM) was employed to establish the MF–EF–IF models of the sugarcane milling system based on the obtained parameters. Moreover, a collaborative optimization model of MF–EF–IF was constructed, and the multi-objective chicken swarm algorithm was studied to determine the optimal value of the process index that makes the whole system tend to be collaborative. Finally, deep reinforcement learning was employed to achieve adaptive optimization of the key operating parameters of the sugarcane milling process and ensure that the system fluctuated around the optimal values.

## 2. Establishment of a Collaborative Optimization Model of MF-EF-IF in the Sugarcane Milling Systems

### 2.1. Sugarcane Milling System

**Figure 1:**
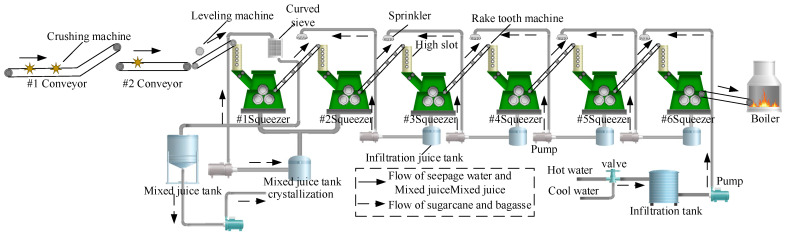
Production process of the sugarcane milling system.

This paper’s research object is the sugarcane milling production process in a sugar factory in Guangxi, China. This system mainly consists of a cane cutter, squeezer, conveyor belt, and permeating water system, as shown in Figure 1. First, the harvested sugarcane is conveyed by two belts and decomposed into filamentous or sheet-like sugarcane material. The sugarcane material is then fed to six sets of squeezers to separate the sugarcane juice and bagasse. During this process, soaking water is added, and the residual sugar is extracted by diffusion, dilution, and displacement caused by the concentration difference between the thin and thick juices.

### 2.2. Analysis of MF-EF-IF in the Sugarcane Milling System

**Figure 2:**
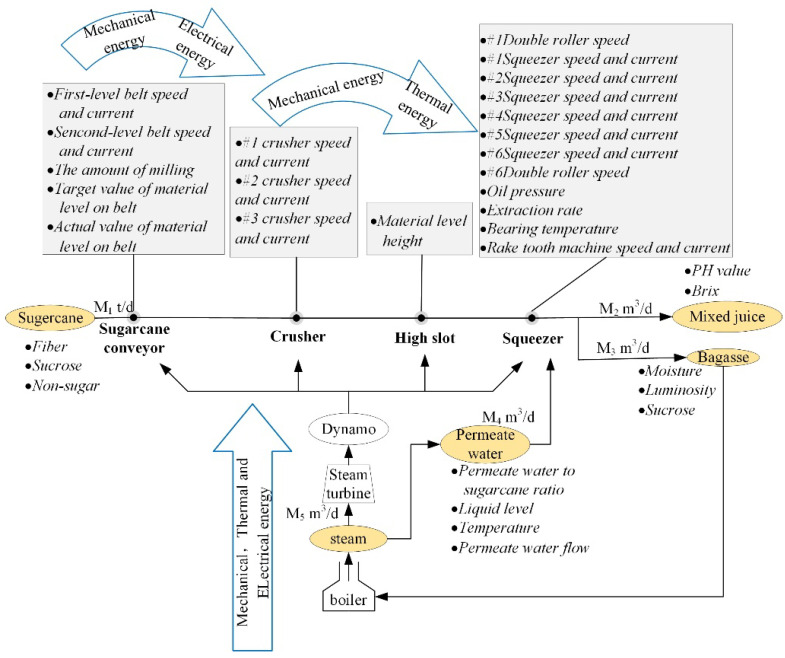
Analysis of material, energy and information flow of the sugarcane milling system.

To better analyze the sugarcane milling system, the process was abstracted as the interaction of material, energy, and information flow (MF–EF–IF) based on the actual production, as shown in Figure 2, which is conducive to analysis of the dynamic and synergistic relationships of the system. The material flow mainly consists of cane material, permeating water, and juice, and is driven by the energy flow, which is composed mainly of electric energy, to produce information flow. In information flow, variables such as the speed of the conveyor and cutter and the ratio of osmotic water to sugarcane are the operating parameters of the sugarcane milling system. Other parameters, including the present load of various machines and the composition of sugarcane, are the working condition parameters of the system. The material flow is the carrier of the energy and information flows, and it is accompanied by the transformation, utilization, and recovery of energy, along with the continuous generation, transmission, and processing of information.

Synergetics notes that the order parameters can dominate the evolution of the process and help the disordered system to transform into a new, ordered structure through self-organization [28]. According to the production objectives of the sugarcane milling process, the order parameters of material, energy, and information flow were determined as the grinding capacity, electric consumption per ton of sugarcane, and sucrose extraction, respectively. The grinding capacity means the amount of sugarcane processed by the milling train per unit of time while maintaining a certain sucrose extraction; the electric consumption per ton of sugarcane indicates the amount of electricity consumed to process 1t of sugarcane material during the milling process; and the sucrose extraction refers to the proportion of the weight of sucrose extracted from the sugarcane during the milling process compared to the total sucrose in the sugarcane. In addition to the order parameters that guide the development trend of the system, the flow also includes parameters that can characterize the state. The state parameters of each flow are usually variables that have a greater impact on the order parameters.

### 2.3. Feature Extraction of MF-EF-IF in the Sugarcane Milling System

**Figure 3:**
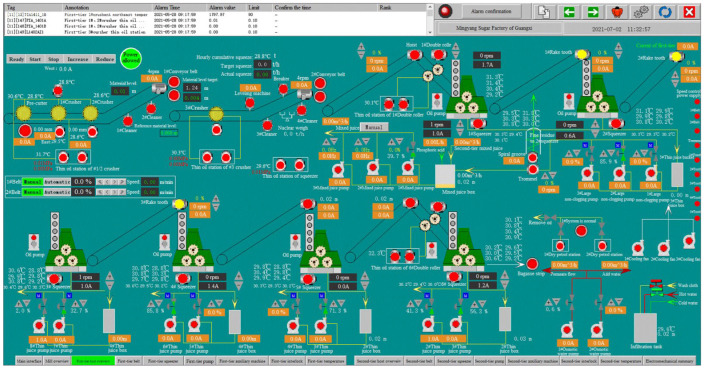
Data monitoring and collection platform of the sugarcane milling system.

In order to obtain the state parameters of each flow, a multilevel filtering method based on mutual information proposed by Meng et al. was used [29]. On the basis of the analysis of the influencing factors of the milling system and the on-site testing data, the operating parameters of the sugarcane milling system were determined, as shown in Table 1. The parameter data were collected by the distributed control system (DCS) installed on the milling system, the interface diagram of which is shown in Figure 3. A total of 1500 sets of operating data from a sugar mill in Guangxi, China, with a sampling interval of 5 min, were used as the research samples.

**Figure 4:**
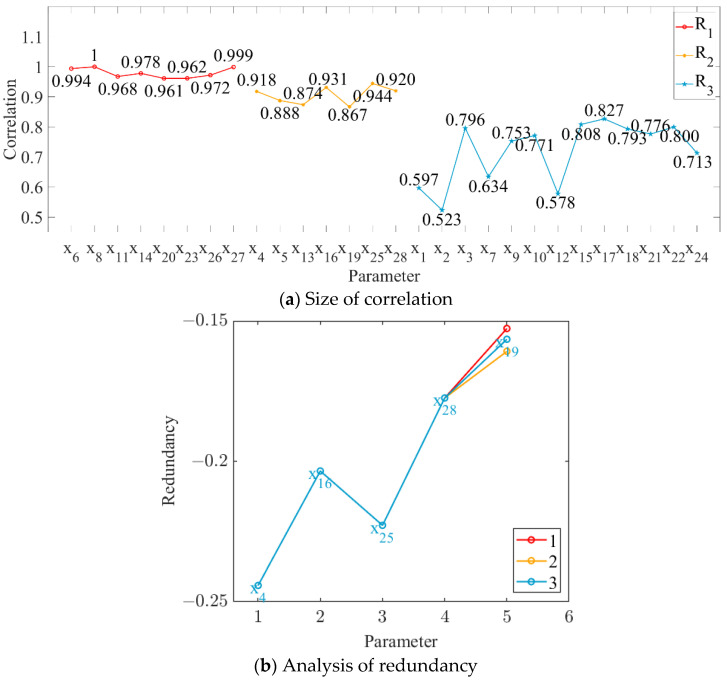
Relationship between parameter variables and grinding capacity.

**Figure 5:**
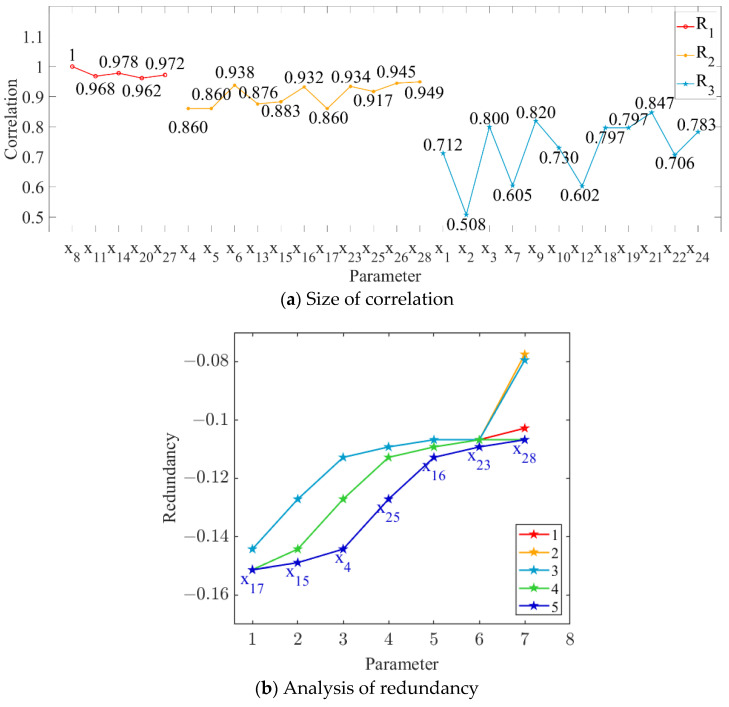
Relationship between parameter variables and electric consumption per ton of sugarcane.

**Figure 6:**
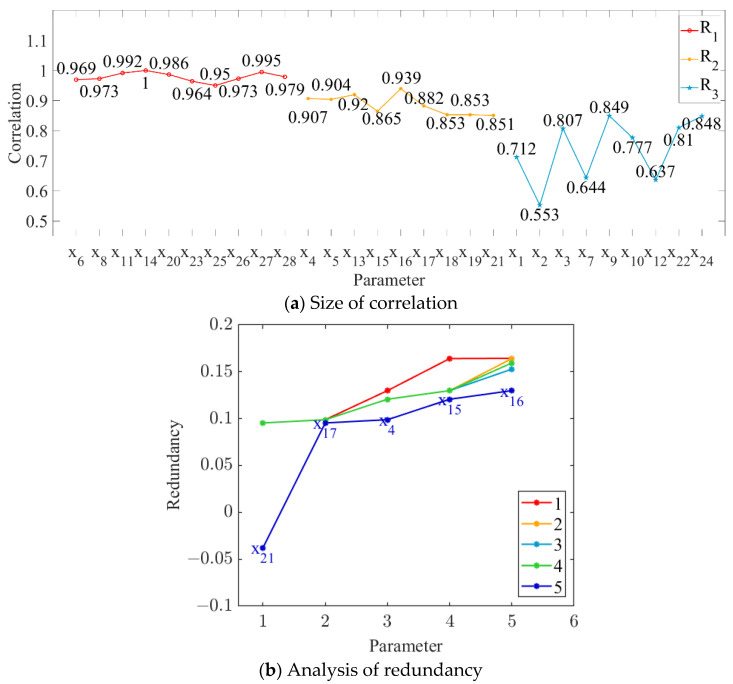
Relationship between parameter variables and sucrose extraction.

In this study, the order parameters (i.e., grinding capacity, electric consumption per ton of sugarcane, and sucrose extraction) were set as the output targets, and the correlation between each parameter and every output target was calculated, as shown in Figure 4a, Figure 5a and Figure 6a. The parameter variables with a correlation degree greater than 0.9 and less than 0.95 are denoted as set M, while those with a correlation degree greater than 0.85 and less than 0.9 are denoted as set N. The parameter variables in set N were sequentially added to set M to obtain the subset Mi. The redundancy degree between subset Mi and each parameter variable with a correlation degree less than 0.85 was calculated, and the feature variables corresponding to the maximum redundancy degree were deleted. The number of redundancy analysis cycles and the results of the final cycle are shown in Figure 4b, Figure 5b and Figure 6b.

As shown in Figure 4, Figure 5 and Figure 6, we deleted the parameter variables corresponding to the maximum redundancy in the final cycle and combined the parameter variables with a correlation greater than 0.95. The feature set with high correlation and low redundancy with order parameters was selected to represent the state parameters of MF–EF–IF. Therefore, #3 crusher current (*x*_4_), first-level belt speed (*x*_6_), second-level belt speed (*x*_8_), #1 squeezer current (*x*_11_), #3 squeezer current (*x*_14_), #6 squeezer current (*x*_20_), #6 double roller current (*x*_23_), permeate water-to-sugarcane ratio (*x*_25_), sucrose content (*x*_26_), non-sugar content of cane (*x*_27_), and cane fiber (*x*_28_) were selected as the state parameters of the material flow; #3 crusher current (*x*_4_), first-level belt speed (*x*_6_), second-level belt speed (*x*_8_), #1 squeezer current (*x*_11_), #3 squeezer current (*x*_14_), #3 squeezer speed (*x*_15_), #4 squeezer current (*x*_16_), #4 squeezer speed (*x*_17_), #6 squeezer current (*x*_20_), #6 double roller current (*x*_23_), permeate water-to-sugarcane ratio (*x*_25_), and non-sugar content of cane (*x*_27_) were selected as the state parameters of the energy flow; and #3 crusher current (*x*_4_), first-level belt speed (*x*_6_), second-level belt speed (*x*_8_), #1 squeezer current (*x*_11_), #3 squeezer current (*x*_14_), #3 squeezer speed (*x*_15_), #4 squeezer speed (*x*_17_), #6 squeezer current (*x*_20_), #6 double roller speed (*x*_21_), #6 double roller current (*x*_23_), permeate water-to-sugarcane ratio (*x*_25_), sucrose content (*x*_26_), non-sugar content of cane (*x*_27_), and cane fiber (*x*_28_) were selected as the state parameters of the information flow.

### 2.4. Establishment of a Collaborative Optimization Model of MF-EF-IF in the Sugarcane Milling Systems

**Figure 7:**
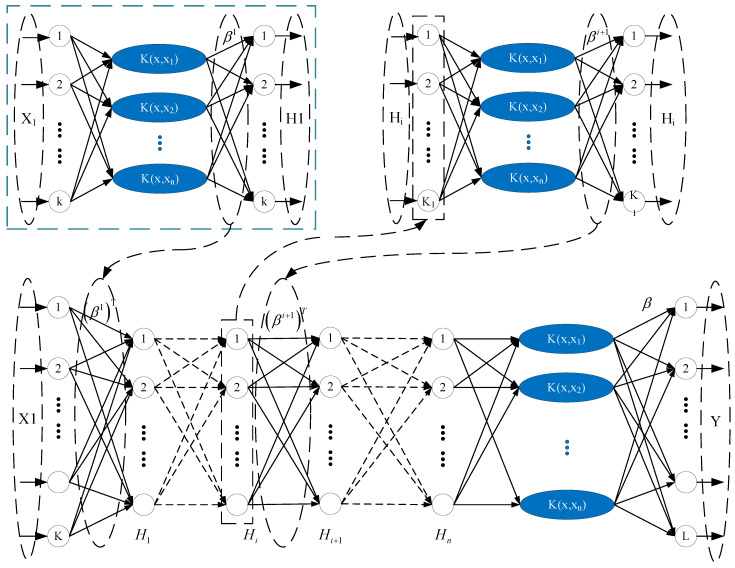
Network structure of the DK-ELM model.

In order to establish the optimal objective function with high fitness with respect to the obtained feature parameters of MF–EF–IF, we combined the kernel extreme learning machine (KELM) proposed by Huang et al. [30], and the kernel extreme learning machine autoencoder (KELM-AE) to propose a deep kernel extreme learning machine (DK-ELM) and construct a data-driven model between the order parameters and the corresponding state parameters; its network structure is shown in Figure 7. This process consisted of two steps: First, the input matrix $X=\left[\begin{array}{llll} x_{11} & x_{12} & \cdots & x_{1d}\\ x_{21} & x_{22} & \ldots & x_{2d}\\ \vdots & \vdots & \ddots & \vdots \\ x_{n1} & x_{n2} & \cdots & x_{nd} \end{array}\right]$ was constructed, and a network of n KELM-AE was used to extract the data features. The weight matrix $\beta_{i}$ of each layer was obtained as follows:

(1) $$\beta_{i}={\left(\frac{I}{C}+\Omega_{ELM}\right)}^{-1}H_{i}$$

(2) $$H_{i+1}=g({\left(\beta^{i+1}\right)}^{T}H_{i})$$

(3) $$\Omega_{}=\left[\begin{array}{cccc} k(x_{1}-x_{1}) & k(x_{1}-x_{2}) & \cdots & k(x_{1}-x_{n})\\ k(x_{2}-x_{1}) & k(x_{2}-x_{2}) & \cdots & k(x_{2}-x_{n})\\ \vdots & \vdots & \ddots & \vdots \\ k(x_{n}-x_{1}) & k(x_{n}-x_{2}) & \cdots & k(x_{n}-x_{n}) \end{array}\right]$$

where K(•) is the kernel function, $H_{i}$ is the output of the *i*-th layer i∈[1,n], g(•) is the activation function, and C is the cost parameter. The stacked network is traversed to calculate each weight matrix $\left[\beta_{1},\;\beta_{2},\cdots \beta_{n-1}\right]$ until the output of the last hidden layer $H_{n}$ is obtained. In the second step, the output of the last hidden layer $H_{n}$ is used as the input of the KELM model, the target set *Y* is the output, and the weight matrix between the hidden layer β and the output layer is solved.

(4) $$\beta ={\left(\frac{I}{C}+\Omega_{ELM}\right)}^{-1}H_{n}$$

(5) $$\Omega_{ELM}=\left[\begin{array}{cccc} k^{\prime }(x_{1}-x_{1}) & k^{\prime }(x_{1}-x_{2}) & \cdots & k^{\prime }(x_{1}-x_{n})\\ k^{\prime }(x_{2}-x_{1}) & k^{\prime }(x_{2}-x_{2}) & \cdots & k^{\prime }(x_{2}-x_{n})\\ \vdots & \vdots & \ddots & \vdots \\ k^{\prime }(x_{n}-x_{1}) & k^{\prime }(x_{n}-x_{2}) & \cdots & k^{\prime }(x_{n}-x_{n}) \end{array}\right]$$

When K(•) is the kernel function, the network output of the DK-ELM is as follows:

(6) $$f_{\mathrm{DK}\_KELM}(X)={\left[\begin{array}{c} K(x,x_{1})\\ \vdots \\ K(x,x_{n}) \end{array}\right]}^{T}{\left(\frac{I}{C}+\Omega_{ELM}\right)}^{-1}Y$$

To better analyze the results of the simulation, the prediction performance is assessed by three indices: the root-mean-square error (RMSE), the mean absolute error (MAE), and the determination coefficient ($R^{2}$). Assuming that the actual value of the *i*-th test sample with m test samples is $y_{i}$, the mean value is $\bar{y}=\frac{\sum_{i=1}^{m}y_{i}}{m}$, and the corresponding data-driven model prediction value is $\overset{\wedge }{y_{i}}$. Each evaluation index formula is as follows:

(7) $$RMSE=\sqrt{\frac{\sum_{i=1}^{m}{\left(y_{i}-{\hat{y}}_{i}\right)}^{2}}{m}}$$

(8) $$MAE=\frac{\sum_{i=1}^{m}\left|y_{i}-{\hat{y}}_{i}\right|}{m}$$

(9) $$R^{2}=\frac{\sum_{i=1}^{m}{\left({\hat{y}}_{i}-{\bar{y}}_{i}\right)}^{2}}{\sum_{i=1}^{m}{\left(y_{i}-{\bar{y}}_{i}\right)}^{2}}$$

This study employs the feature combination of each flow subsystem as the inputs and takes the grinding capacity, electric consumption per ton of sugarcane, and sucrose extraction as the outputs, and the sugarcane milling system’s MF–EF–IF models are constructed based on DK-ELM. Among them, the kernel function is selected as the Gaussian radial basis function $K\left(x,x_{i}\right)=\exp({\left\|x-x_{i}\right\|}^{2}/\gamma )$, with wide applicability and only one parameter variable. The sigmoid function is selected as the activation function. The parameters that need to be adjusted are the penalty factor *C* and kernel function parameters γ. The number of hidden layers and the hyperparameters for each layer of the constructed DK-ELM model affect the model’s accuracy. At the same time, the feature extraction method described in Section 2.3 only considers the effects of individual features on the output target, and the effects of different feature combinations on the model are not considered. In order to obtain better performance, the wrapper method based on the improved chicken swarm optimization (ICSO) proposed by Meng et al. is used to obtain the optimal parameter combinations [29]. The training model’s determination coefficient ($R^{2}$) is taken as the fitness, and a combination of the ICSO and trial-and-error methods is used to optimize the hyperparameters. The number of hidden layers is sequentially accumulated to obtain the optimal parameters until the fitness function no longer increases. The range of parameter optimization is set to [0.01, 1000], and the model parameter results after iteration are shown in Table 2.

As the iterations end, the optimal feature combinations of the material, energy, and information flows are obtained as {*x*_6_, *x*_8_, *x*_11_, *x*_14_, *x*_20_, *x*_26_, *x*_28_}, {*x*_4_, *x*_8_, *x*_14_, *x*_15_, *x*_20_, *x*_23_, *x*_25_, *x*_27_}, and {*x*_4_, *x*_8_, *x*_11_, *x*_14_, *x*_17_, *x*_20_, *x*_21_, *x*_23_, *x*_25_, *x*_27_}, respectively, as shown in Table 2. With the optimal combination of MF–EF–IF as the inputs and the corresponding order parameters (grinding capacity $y_{1}$, electric consumption per ton of sugarcane $y_{2}$, and sucrose extraction $y_{3}$) as outputs, using the obtained optimal combination of features and hyperparameters, the data-driven model of the sugarcane milling system is constructed and can be expressed as follows:

(10) $$\begin{array}{l} y_{1}=f_{1}(x_{6},x_{8},x_{11},x_{14},x_{20},x_{26},x_{28})\\ y_{2}=\;f_{2}(x_{4},x_{8},x_{14},x_{15},x_{20},x_{23},x_{25},x_{27})\\ y_{3}=\;f_{3}(x_{4},x_{8},x_{11},x_{14},x_{17},x_{20},x_{21},x_{23},x_{25},x_{27}) \end{array}$$

With the optimization goals of maximizing the grinding capacity and sucrose extraction and minimizing the electric consumption in the squeezing process, using the constructed data-driven model as the fitness function, the collaborative optimization model of MF–EF–IF in the sugarcane milling process can be constructed based on the range value of each input variable of the DK-ELM model presented in Table 3, as shown in Equation (11):

(11) $$\begin{array}{l} Max\;\;\;\;\;\;f_{1}(x_{6},x_{8},x_{11},x_{14},x_{20},x_{26},x_{28})\\ Min\;\;\;\;\;\;f_{2}(x_{4},x_{8},x_{14},x_{15},x_{20},x_{23},x_{25},x_{27})\\ Max\;\;\;\;\;f_{3}(x_{4},x_{8},x_{11},x_{14},x_{17},x_{20},x_{21},x_{23},x_{25},x_{27})\\ s.t.\;\;\;\;\;\;\begin{array}{cc} 53\leq x_{4}\leq 66; & 631\leq x_{20}\leq 804 \end{array}\\ \;\;\;\;\;\;\;\;\;\;\;\;\;\begin{array}{cc} 5.5\leq x_{6}\leq 8.1; & 3.3\leq x_{21}\leq 4.2 \end{array}\\ \;\;\;\;\;\;\;\;\;\;\;\;\;\begin{array}{cc} 6.1\leq x_{8}\leq 7.8; & 906\leq x_{23}\leq 1092 \end{array}\\ \begin{array}{cc} \;\;\;\;\;\;\;\;\;\;\;\;\;969\leq x_{11}\leq 1092; & 15.94\leq x_{25}\leq 21.72 \end{array}\\ \;\begin{array}{cc} \;\;\;\;\;\;\;\;\;\;\;\;788\leq x_{14}\leq 934; & 14.19\leq x_{26}\leq 14.91 \end{array}\\ \begin{array}{cc} \;\;\;\;\;\;\;\;\;\;\;\;\;4.9\leq x_{15}\leq 6.2; & 2.16\leq x_{27}\leq 2.59 \end{array}\\ \;\begin{array}{cc} \;\;\;\;\;\;\;\;\;\;\;\;\;3.5\leq x_{17}\leq 5.6; & 10.16\leq x_{28}\leq 10.5 \end{array} \end{array}$$

## 3. Solving the Collaborative Optimization Model of MF-EF-IF in the Sugarcane Milling System

### 3.1. Multi-Objective Chicken Swarm Optimization Solution Strategy Based on Flow Collaboration

In order to solve the collaborative optimization model constructed in Section 2.4 and investigate the optimal process indicators to guide the adjustment of the operating parameters, a multi-objective chicken swarm optimization algorithm based on flow collaboration (IMOCSO) is proposed in this paper. The specific contents include the following:

(1) Hierarchical relationship update between chicken populations: In the MOCSO, the synergy degree (SE) is selected as the aggregation function of multiple objectives. The MOCSO algorithm sorts the population of chickens according to the values of the aggregate objective function and follows the rate into the rooster (NR), hen (NH), and chick (NC) population groups. The order parameters are discussed with two opposite effects: The positive effect means that the degree of order of the subsystem increases as the order parameter increases [31]. Conversely, a negative effect means that the degree of order of the subsystem decreases as the order parameter increases [32]. Based on the efficacy coefficient, the degree of synergy among the MF–EF–IF can be introduced to show the overall performance of the milling system. The efficacy coefficient (*Fs*) and synergy degree (*SE*) of the order parameter are calculated as follows:

(12) $$Fs(\mu_{i})(i=1,2,3)=\{\begin{array}{c} \frac{\max(\mu_{i})-\mu_{i}}{\max(\mu_{i})-\min(\mu_{i})}(negative\;effect)\\ \frac{\mu_{i}-\min(\mu_{i})}{\max(\mu_{i})-\min(\mu_{i})}(positive\;effect) \end{array}$$

(13) $$SE=\sqrt[{3}]{Fs(\mu_{1})\cdot Fs(\mu_{2})\cdot Fs(\mu_{3})}$$

where $\mu_{i}$ is the order parameter, *i* is the *i*-th flow, and $\max(\mu_{i})$ and $\min(\mu_{i})$ are the maximum and the minimum of $\mu_{i}$, respectively.

(2) Update the position of each chicken group: The forward learning mechanism is introduced into the rooster subgroup, which can accelerate the rate of convergence.

(14) $$x^{t+1}{}_{i}=x^{t}{}_{i}*(1+Randn(0,\sigma^{2}))+w_{1}(x^{t}{}_{best}-x^{t}{}_{i})$$

(15) $$\sigma^{2}=\left\{ \begin{array}{l} 1\;\;\;\;\;\;\;\;\;\;\;\;\;\;\;\;\;\;\;\;\;\;\;\;if\;x_{i}\underline{≺}\;x_{k}\\ \exp(\frac{SE_{k}-SE_{i}}{\left|SE_{i}\right|\;+\;\varepsilon }),otherwise \end{array}\right.$$

where $\;x_{i}\underline{≺}\;x_{k}$ indicates that the *i*-th rooster weakly dominates the *k*-th rooster, $Randn(0,\sigma^{2})$ is a Gaussian distribution with a mean of zero and a standard deviation of $\sigma^{2}$, ε is a small constant to prevent the denominator from being zero, $x^{t}{}_{i}$ is the position of the *i*-th rooster at the *t*-th iteration, $x^{t+1}{}_{i}$ is the position of the *i*-th rooster at the t + 1-th iteration, and $x^{t}{}_{best}$ is the globally optimal individual at the *t*-th iteration, which has the largest degree of collaboration in the archive, while $w_{1}$ is the learning factor of forward learning. According to Equations (12) and (13), the *SE* of each rooster is calculated, where $SE_{i}$ is the synergy degree of the *i*-th rooster, and $SE_{k}$ is the synergy degree of the *k*-th individual. The hen randomly selects the rooster to follow, and its position is updated as follows:

(16) $$x^{t+1}{}_{i}=x^{t}{}_{i}+S_{1}*rand*(x^{t}{}_{r1}-x^{t}{}_{i})+S_{2}*rand*(x^{t}{}_{r2}-x^{t}{}_{i})$$

(17) $$S_{2}=\exp(SE_{r_{2}}-SE_{i})$$

(18) $$S_{1}=\exp(\frac{SE_{i}-SE_{r_{1}}}{\left|SE_{i}\right|+\varepsilon })$$

where $x^{t}{}_{i}$ is the position of the *i*-th hen at the *t*-th iteration, $x^{t+1}{}_{i}$ is the position of the *i*-th hen at the *t* + 1-th iteration, $x^{t}{}_{r1}$ is the rooster followed by the *i*-th hen at the *t*-th iteration, $x^{t}{}_{r2}$ is the rooster or hen randomly selected from the whole flock, and $r_{1}\neq r_{2}$; $SE_{i}$, $SE_{r1}$, and $SE_{r2}$ are the synergy degree of the *i*-th, *r*1-th, and *r*2-th individuals, respectively. The parental guidance mechanism and adaptive factors are introduced into the chick’s position update as follows:

(19) $$x^{t+1}{}_{i}=w*x^{t}{}_{i}+\lambda_{1}*(x^{t}{}_{m}-x^{t}{}_{i})+\lambda_{2}*(x^{t}{}_{r1}-x^{t}{}_{i})$$

where $x^{t}{}_{i}$ is the position of the *i*-th chick at the *t*-th iteration, $x^{t+1}{}_{i}$ is the position of the *i*-th chick at the *t* + 1-th iteration, $x^{t}{}_{m}$ is the hen followed by the *i*-th individual, $x^{t}{}_{r_{1}}$ is the rooster followed by the *i*-th chick, w is the weight, and $\lambda_{1}$ and $\lambda_{2}$ are the learning factors from the hens and roosters, respectively.

(3) Maintenance of external archives: The obtained non-dominated solution set is stored in an external archive. An exponential function is introduced to maintain information sharing between the particles to avoid the explosion of—And preserve the diversity of—The archive population. The Euclidean distance $d_{ij}$ is used to measure the degree of aggregation between the *i*-th particle and the *j*-th particle, after which an exponential distance update is introduced [33].

(20) $$d_{ij}=\left\|x_{i}-x_{j}\right\|=\sqrt{\sum_{k=1}^{n}{\left(x_{i,k}-x_{j,k}\right)}^{2}}$$

(21) $$x^{t}{}_{i,k}=\left(x^{t}{}_{i,k}-\frac{uniformrnd\left(Lb_{k},Ub_{k}\right)}{{\left(Ub_{k}-Lb_{k}\right)}^{2}}\right)*\frac{e^{-\left(x^{t}{}_{i,k}-\frac{uniformrnd\left(Lb_{k},Ub_{k}\right)}{{\left(Ub_{k}-Lb_{k}\right)}^{2}}\right)}}{{\left(Ub_{k}-Lb_{k}\right)}^{2}}$$

where $d_{ij}$ is the distance between the *i*-th particle and the *j*-th particle, $Ub_{k}$ and $Lb_{k}$ are the upper and lower limits of the *k*-th variable, respectively, and the function uniformrnd() represents a randomly selected normal distribution value.

**Figure 8:**
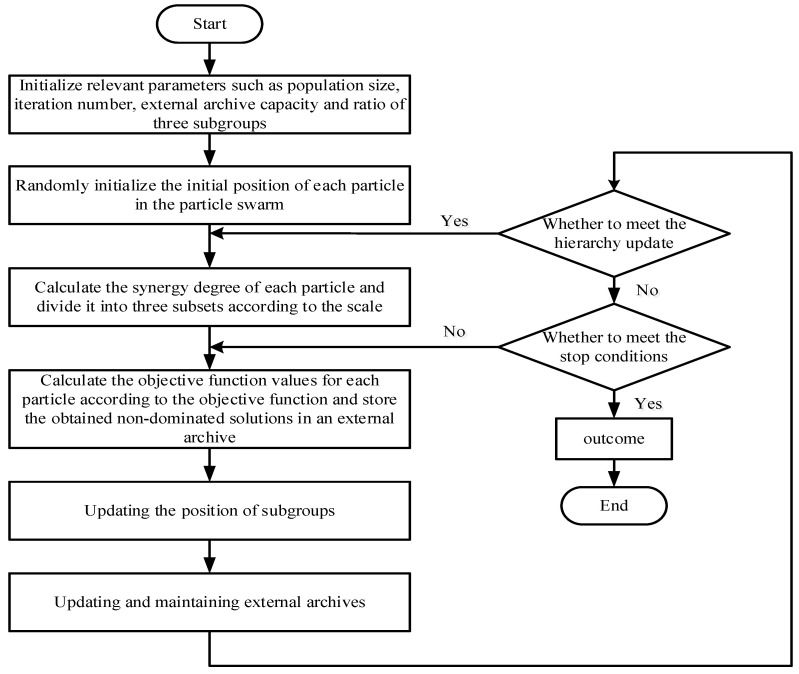
Solution strategy of multi-objective chicken swarm optimization based on flow collaboration.

The IMOCSO algorithm is used to solve the established model, and the solution process is shown in Figure 8.

### 3.2. Adaptive Optimization of Operating Parameters Based on Deep Reinforcement Learning

The sugarcane milling process is a 24/7 production process. During the production, when the order parameters of the sugarcane milling process (i.e., grinding capacity, electric consumption per ton of sugarcane, and sucrose extraction) fluctuate, it is necessary to adjust the operating parameters so that the order parameters can be quickly return to near the optimal target. Due to the working conditions are constantly changing throughout the production process, the operating parameters need to be continuously adjusted during the production cycle to make the production process stable. Therefore, adaptive optimization of the sugarcane milling process means that the operational parameters are continuous adjusting according to the real-time detection values of the order parameters when the working conditions of the production process change, ensuring that the order parameters are stable in the optimal range. 

In Section 2.4, a data-driven model of MF–EF–IF model is presented for real-time detection values of the order parameters, while in Section 3.1 MOCSO is used to solve the optimal values of the order parameters under all working conditions. However, there are many contradictions among the order parameters, and constraints such as production boundary conditions will change with time, resulting in the optimal solution set and Pareto frontier surface also changing with time. The traditional multi-objective optimization methods has been unable to adapt to the new production environment, and it is difficult to quickly track the Pareto frontier and Pareto solution set after detecting the environmental changes. Therefore, on the basis of the above, a deep reinforcement learning technique was introduced and applied to the sugarcane milling process to optimize the process’ operating parameters.

Deep reinforcement learning (DRL) is a technique to train an agent to interact with its environment and to learn the mapping relationship from state to behavior based on the powerful fitting capability of a neural network. DRL uses the Markov decision process (MDP) to model the training process, including four basic elements: M=(S,A,P,R), where S is the set of all states of the process, A is the set of all possible actions taken, P denotes the probability of the occurrence of a transfer from one state to another, and $R:S\times A\to [-R_{\max},R_{\max}]$ is the reward function by which the action taken by the agent affects the environmental state. Li et al. developed a deep-reinforcement-learning-based online path-planning approach for unmanned aerial vehicles (UAVs) and used Markov decision processes to define and explain the UAV state space, UAV action space, and reward functions [34]. Zhang et al. proposed a deep-reinforcement-learning-based energy scheduling strategy to optimize multiple targets, taking diversified uncertainties into account; an integrated power, heat, and natural gas system consisting of energy-coupling units and wind power generation interconnected via a power grid was modeled as a Markov decision process [35]. Liu et al. proposed an adaptive uncertain dynamic economic dispatch method based on deep deterministic policy gradient (DDPG); on the basis of the economic dispatch model, they built a Markov decision process for power systems [36]. In this paper, the operation optimization of the sugarcane milling process is described as an MDP process, which is modeled as follows:

(1) State space S: The state space determines the environmental perception of the agent. On the basis of the obtained state parameters of MF–EF–IF of the milling system as described in Section 2.3, 14 parameters with a certain influence on the order parameters—Such as #2 crusher current (West) (*x*_3_) and #3 crusher current (*x*_4_)—Are selected as the state space. The state space is expressed as follows:

(22) $$S_{t}=\{x_{3},x_{4},x_{6},x_{7},x_{13},x_{14},x_{16},x_{19},x_{21},x_{25},x_{26},x_{27},x_{28}\}$$

(2) Action space A: The action space of the agent is the algorithm’s output, which comprises the operating parameters that need to be adaptively adjusted. Based on the principle that the selection of action should be consistent with the actual control variables, the key process parameters of the sugarcane milling process—I.e., first-level belt speed (*x*_6_), second-level belt speed (*x*_8_), #3 squeezer speed (*x*_15_), #4 squeezer speed (*x*_17_), and #6 double roller speed (*x*_21_)—Are selected as the action space. Assuming that the speed control of the first five actions is v_1_, v_2_, v_3_, v_4_, and v_5_, respectively, and that the control action of osmotic water on the sugarcane ratio is h, the action space is expressed as follows:

(23) $$a_{t}=\{v_{1},v_{2},v_{3},v_{4},v_{5},h\}$$

(3) Reward function R(s,a): The agent evaluates the action taken by the reward function. Considering that the optimization objective is to minimize the deviation between the optimal values of the order parameters obtained in Section 3.1 (i.e., grinding capacity, electric consumption per ton of sugarcane, and sucrose extraction) and their actual values, the reward function (R(s,a)) of different actions under different states is determined as follows:

(24) $$R(s,a)=\sum_{i=1}^{3}\frac{\sqrt{(f_{i}(s_{t})-p_{i}{)}^{2}}}{3}-\sum_{i=1}^{3}\frac{\sqrt{(f_{i}(s_{t+1})-p_{i}{)}^{2}}}{3}$$

where $f_{i}$ is the mathematical model of MF–EF–IF based on the DK-ELM method, and $p_{i}$ represents the optimal order parameters of each flow solved by the MOCSO based on flow collaboration.

It is necessary to choose a specific depth-enhanced learning framework combining the application scenarios of each algorithm along with its advantages and disadvantages. Common deep reinforcement learning methods include deep Q networks (DQNs), Actor–Critic (AC), policy gradient (PG), and deep deterministic policy gradient (DDPG) [37,38,39]. Considering that the optimization of the operational parameters in sugarcane milling is a continuous process, the DDPG algorithm composed of an actor–critic framework is selected. After DDPG perceives the environmental state $s_{t}$, the actor online policy network outputs the action $a_{t}=\mu (s_{t}|\theta^{\mu })$, and the critic online Q network evaluates the action value $Q=Q(s_{t},a_{t}|\theta^{Q})$, where $\theta^{\mu }$ and $\theta^{Q}$ are the actor and critic online network parameters, respectively. In order to improve the stability of the algorithm, the actor target policy network and target Q network are also constructed.

To update the actor and critic networks, DDPG draws N small batches of sequence data $\left\{s_{t},a_{t},r_{t+1},s_{t+1}\right\}$ from the experience playback pool M to train the model, and the critic network is updated in the direction of the minimization loss function *L*, denoted as follows:

(25) $$L(\theta^{Q})\approx \frac{1}{N}{\sum_{i=1}^{N}\left[y_{i}-Q(s_{{}^{i}},a_{{}^{i}}|\theta^{Q})\right]}^{2}$$

where $y_{i}=r_{i}+\gamma Q^{\prime }(s_{i+1},\mu^{\prime }(s_{i+1}|\theta^{\mu^{\prime }})|\theta^{Q^{\prime }})$ is the target value, *i* is the extracted sample sequence number, γ∈[0,1] is the discount factor, $\mu^{\prime }(s_{i+1}|\theta^{\mu^{\prime }})$ is the determined action of the target policy network based on the output of the next state $s_{i+1}$, and $\theta^{\mu^{\prime }}$ and $\theta^{Q^{\prime }}$ represent the parameters of the actor target policy network and the target Q network, respectively. Meanwhile, the actor network is updated according to the policy gradient as follows:

(26) $$\nabla_{\theta^{\mu }}J=\frac{1}{N}\sum_{i=1}^{N}[\nabla_{a}Q(s,a|\theta^{Q})|{}_{s=s_{i},a=\mu (s_{i}|\theta )}\nabla_{\theta^{\mu }}\mu (s|\theta^{\mu })|{}_{s=si}]$$

The parameters of the target valuation network and the target policy network in DDPG are updated in a soft manner, as follows:

(27) $$\theta^{\mu^{\prime }}=\tau \theta^{\mu^{\prime }}+(1-\tau )\theta^{\mu^{\prime }},0<\tau <<1$$

(28) $$\theta^{Q^{\prime }}=\tau \theta^{Q^{\prime }}+(1-\tau )\theta^{Q^{\prime }},0<\tau <<1$$

Due to the introduction of the soft update method, the parameters of the target network are updated by a smaller magnitude each time, making it easier to converge and more stable.

In order to ensure that the diversity of samples in the experience pool is conducive to network convergence, a random discarding sample based on the sample similarity algorithm is introduced during the network training to improve the DDPG algorithm. Sample similarity is calculated as follows:

(29) $$sim(x_{i}{}^{*},x_{ki})=1-\frac{\left|x_{i}{}^{*}-x_{ki}\right|}{\max(x_{i}{}^{*},x_{ki})}$$

where $sim(x_{i}{}^{*},x_{ki})$ is the sample similarity, $x_{i}{}^{*}$ is the state space of the running process, $x_{ki}$ is the state space in the sample pool, |•| is the Euclidean distance between $x_{i}{}^{*}$ and $x_{ki}$, and max() is the maximum of all Euclidean distances; the greater the similarity, the higher the probability of discarding that sample.

**Figure 9:**
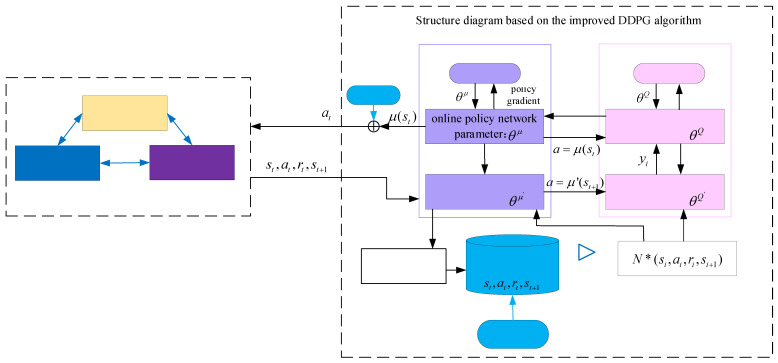
An optimization framework of operation parameters for sugarcane milling process based on improved DDPG.

The optimization framework of the operating parameters in the sugarcane milling process based on improved DDPG is shown in Figure 9, and the improved DDPG algorithm is used to realize the adaptive adjustment of operating parameters in the sugarcane milling process, which is solved in the following steps:

Step 1: First, the experience pool *D* with capacity *N*, the action value network, and the policy network are initialized, and the weight parameters are randomly generated. Then, the parameters of the action value network and the policy network are initialized and copied to the corresponding target network;

Step 2: The Ornstein–Uhlenbeck *(OU)* noise of the random process for action exploration is initialized, and the current state $S_{t}$ is obtained. The action is selected based on the current policy network and noise, and then the current action $a_{t}$ is executed to update the environment and to obtain the rewards $r_{t}$ and the next moment state $S_{t+1}$;

Step 3: The sample similarity between the current state space and the state space in the experience pool is calculated. The state is discarded if the similarity is greater than a given threshold; otherwise, it is stored in the experience pool. Step 3 is repeated to determine whether the inner loop is reached; if so, Step 2 is repeated;

Step 4: After a certain number of data are stored in the experience pool, a small batch of trajectory data $\left\{s_{t},a_{t},r_{t+1},S_{t+1}\right\}$ are randomly sampled from the experience pool *D* at specific time intervals. The target action value network and the policy network are updated according to Equations (25) and (26), and the action value network and policy network are softly updated after a certain time interval;

Step 5: The above steps are repeated until the training times are achieved, and the set values of the optimal process parameters are output.

## 4. A Framework of Optimization for Sugarcane Milling System Operation

**Figure 10:**
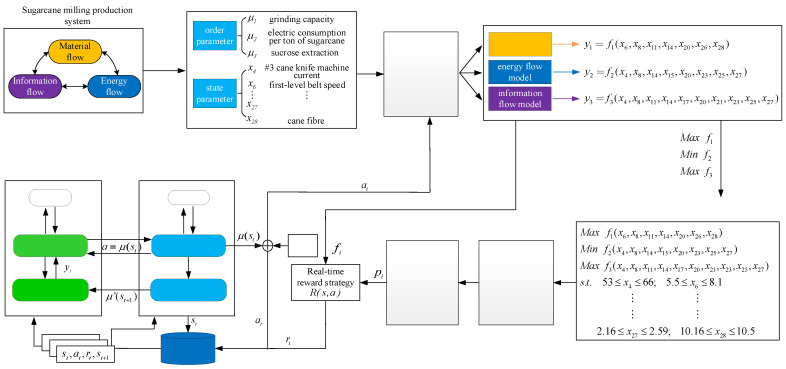
Operation optimization framework of the sugarcane milling system.

In order to achieve the global optimization of the milling process, the sugarcane milling process is abstracted as a system with the interaction of MF–EF–IF, and the proposed optimization framework of the sugarcane milling system, as shown in Figure 10, includes establishing a collaborative optimization model of MF–EF–IF and the solution of the optimization model. The state features of MF–EF–IF are obtained by combining mutual information and a hybrid chicken swarm optimization algorithm. Then, a data-driven model of MF–EF–IF is established by using the DK-ELM method. With the optimization objectives of minimizing electric consumption per ton of cane and maximizing the grinding capacity and sucrose extractions, a collaborative optimization model of MF–EF–IF is constructed. The solution of the optimization model is composed of two parts: In the first part, the MOCSO algorithm based on flow collaboration is used to solve the optimal values of the order parameters under all working conditions, providing guidance for optimizing the operational parameters. In the second part, the optimal values of the obtained order parameters are selected as the control objectives, the optimal operation parameter setting values under different working conditions are determined based on the trained DDPG model, and the adaptive adjustment of the whole process is realized.

## 5. Experimental Results and Discussion

### 5.1. Result and Analysis of the Data-Driven Model of MF-EF-IF in the Sugarcane Milling System

**Figure 11:**
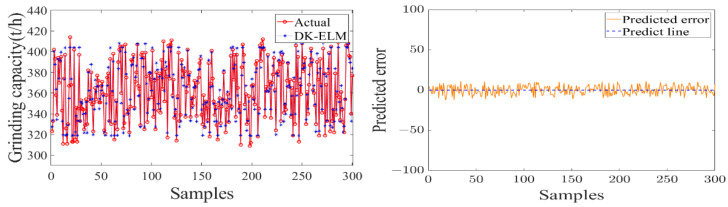
Modeling result of material flow with grinding capacity as model output.

**Figure 12:**
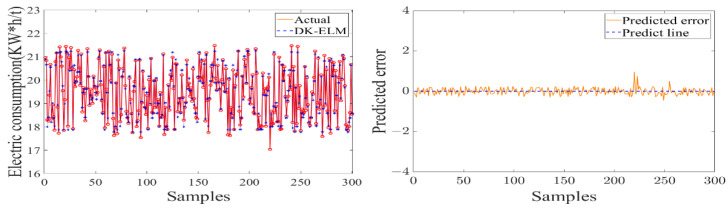
Modeling result of energy flow with electric consumption per ton of sugarcane as model output.

**Figure 13:**
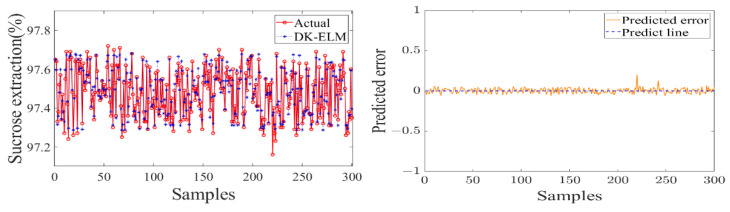
Modeling result of information flow with sucrose extraction as model output.

In order to study the data-driven modeling, the used sample set was kept consistent with the feature extraction. The dataset was randomly divided into five equal parts, of which four equal parts were used as the training set to construct a data-driven model of the sugarcane milling process and to determine the optimal combination of features and hyperparameters of the model. The remaining data were used as the test set to verify the model’s output accuracy and degree of fit. The performance evaluation of each flow subsystem model is shown in Table 4. Figure 11, Figure 12 and Figure 13 show the test results and errors of the data-driven models of material, energy, and information flows, respectively.

As shown in Table 4, the training of the data-driven model of MF–EF–IF of the sugarcane milling system takes less than 1 s, indicating that it has a fast learning speed. Secondly, the values of the evaluation metrics (RMSE and MAE) are small and fluctuate within the acceptable range, with *R*^2^ of 0.9569, 0.9776, and 0.9282, respectively. This indicates that the model has a high degree of fit and good learning performance. As shown in Figure 11, Figure 12 and Figure 13, the constructed data-driven model has a good ability to predict the grinding capacity, electric consumption per ton of sugarcane, and sucrose extraction. The predicted values are very close to the actual values, and there are no data points with substantial errors. The error curve fluctuates smoothly and around zero, indicating that the model has good generalization performance and can be used for modeling the MF–EF–IF of the sugarcane milling process.

### 5.2. Obtaining the Optimal Values of the Order Parameter

In order to study the optimal performance of the sugarcane milling process under all working conditions, the grinding capacity, electric consumption per ton of sugarcane, and sucrose extraction were used as objective functions, and the IMOSCO algorithm proposed in Section 3.1 based on the implemented DK-ELM model was used for that purpose. The parameter settings of the IMOSCO algorithm are summarized in Table 5. 

**Figure 14:**
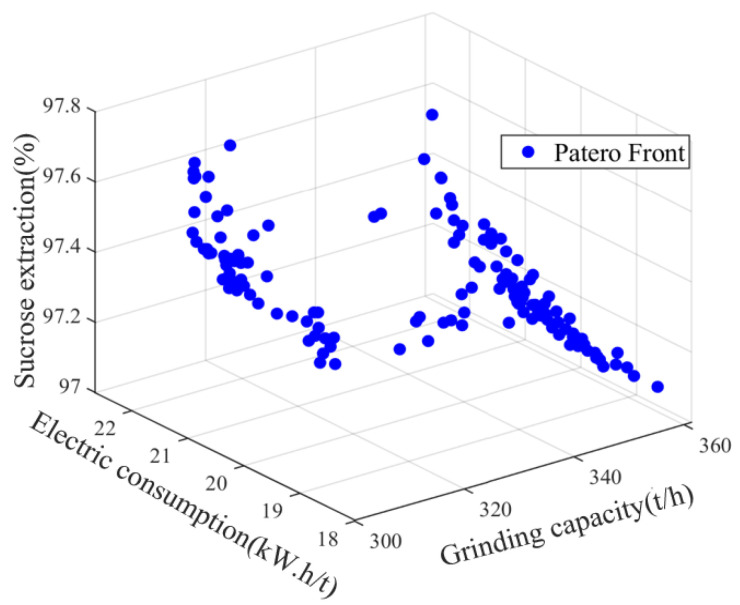
Pareto fronts for order parameters of material, energy and information flow.

**Figure 15:**
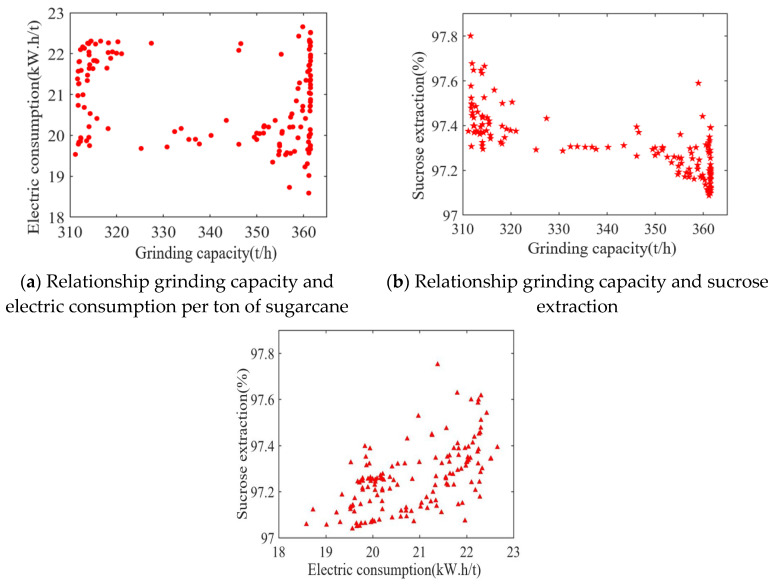
Relationship between the three objectives. (**a**) Relationship grinding capacity and electric consumption per ton of sugarcane. (**b**) Relationship grinding capacity and sucrose extraction. (**c**) Relationship electric consumption per ton of sugarcane and sucrose extraction.

The results of the Pareto solution set and the relationships between the different objectives are shown in Figure 14 and Figure 15. Among them, the maximum grinding capacity is 370 t/h, the minimum grinding capacity is 310 t/h, the maximum electric consumption per ton of sugarcane is 22.7 kW.h/t, the minimum electric consumption per ton of sugarcane is 18.5 kW.h/t, the maximum sucrose extraction is 97.8%, and the minimum sucrose extraction is 97%. As visualized in Figure 14, the reduction in the electric consumption per ton of sugarcane leads to a reduction in the sucrose extraction, and there is a contradiction between the two objective functions. At the same time, as demonstrated in Figure 15, it is not the case that a greater grinding capacity will lead to a higher sucrose extraction. Meanwhile, with the increase in the grinding capacity, the electric consumption per ton of sugarcane will also increase, because the speed of the milling process will increase to meet the actual production demand as the amount of feed in the mill process increases, and there will be more energy loss as a result. Therefore, the contradictions between these three objective functions are consistent with the relationships in the actual sugarcane milling system. To find the global optimum, the particles in the archive are sorted according to their degree of maximum synergy, where the optimal point is at a grinding capacity of 353.38 t/h, an electric consumption per ton of sugarcane of 17.09 kW.h/t, and an sucrose extraction of 97.87%. The obtained set of values is chosen as the global optimal index to guide the adjustment of the process parameters.

### 5.3. Results of the Operation Optimization of the Operating Parameters in the Sugarcane Milling Process

#### 5.3.1. DDPG Parameter Settings and Optimization Results

Based on the optimal values of the order parameters, the improved DDPG algorithm was applied to the optimization of key operating parameters in the sugarcane milling process, and the relevant training parameter settings of DDPG are shown in Table 6. The optimal solution of the order parameters was set as the optimal goal of the experiment (the grinding capacity was set at 353.38 t/h, the electric consumption per ton of sugarcane was set at 17.09 kW.h/t, and the sucrose extraction was set at 97.87%), and the neural network parameter settings of DDPG are demonstrated in Table 7.

**Figure 16:**
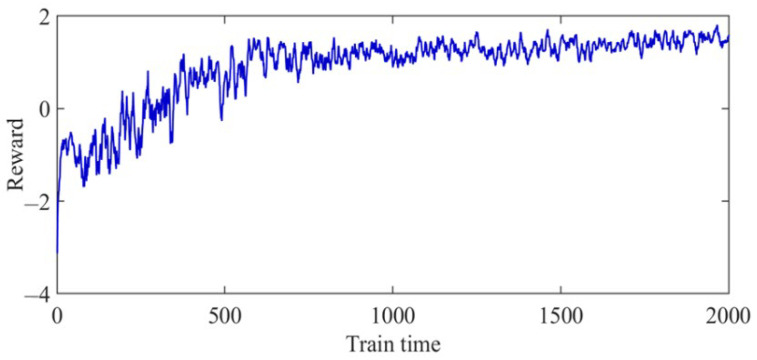
Error value of agent training.

First, the agent was trained 2000 times, and then, the reward value obtained by the agent was recorded at each iteration time in the training process of the DDPG algorithm. The training results are shown in Figure 16. The obtained reward value fluctuated up and down before 1000 iterations of the training process; that is, the agent was constantly learning how to deal with the newly generated working conditions, and the parameters of each network were also in a continuous process of adjustment and optimization. After 1000 iterations of the training process, the reward value obtained tended to be stable, indicating that the decision-making ability of the agent was significantly improved. 

**Figure 17:**
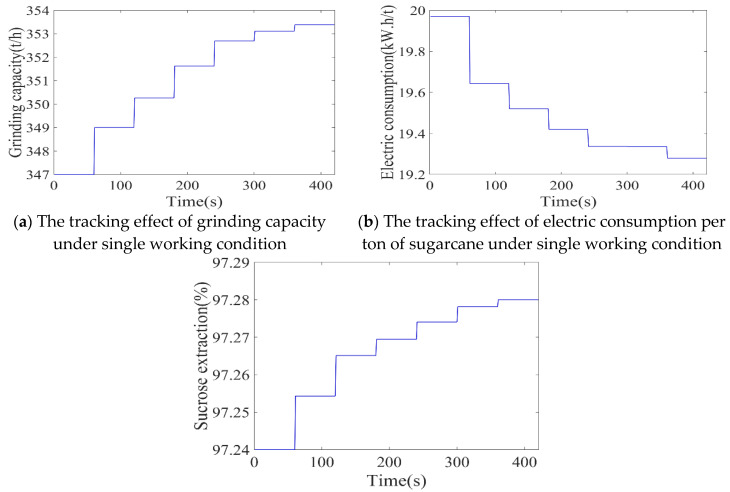
Each control target following curve of a single working condition.

After 2000 iterations of training, the trained DDPG model was used for different working conditions of the sugarcane milling production process. The tracking effect of the agent under specific working conditions is shown in Figure 17. The trained agent can adaptively determine the optimal setting strategy according to the current working condition. The final tracking grinding capacity was 353.38 t/h, the electric consumption per ton of sugarcane was 17.09 kW.h/t, and the sucrose extraction was 97.87%, all of which fluctuated near the optimal index.

**Figure 18:**
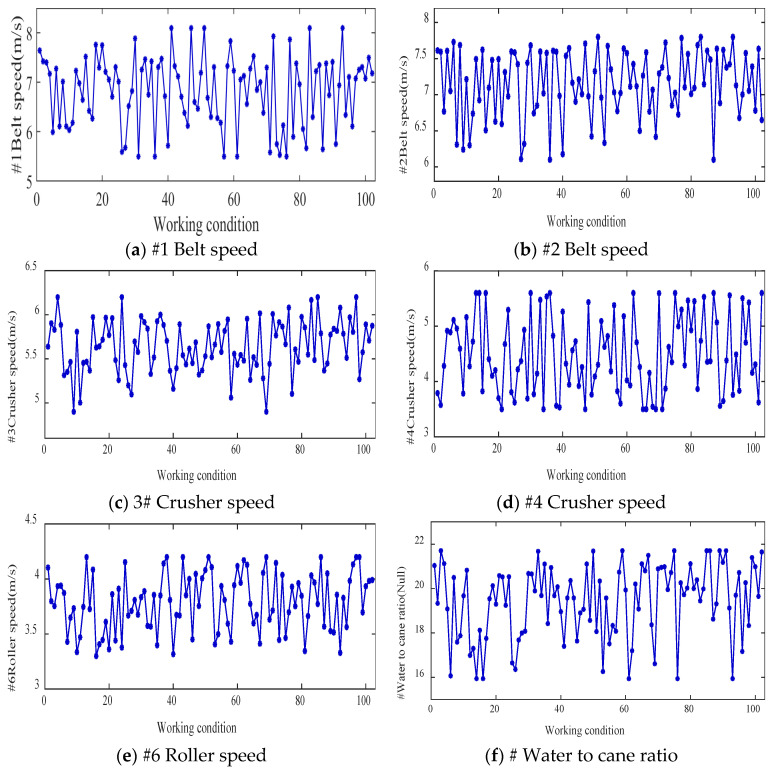
Optimal setting of operation parameters under different working conditions in sugarcane milling production process.

**Figure 19:**
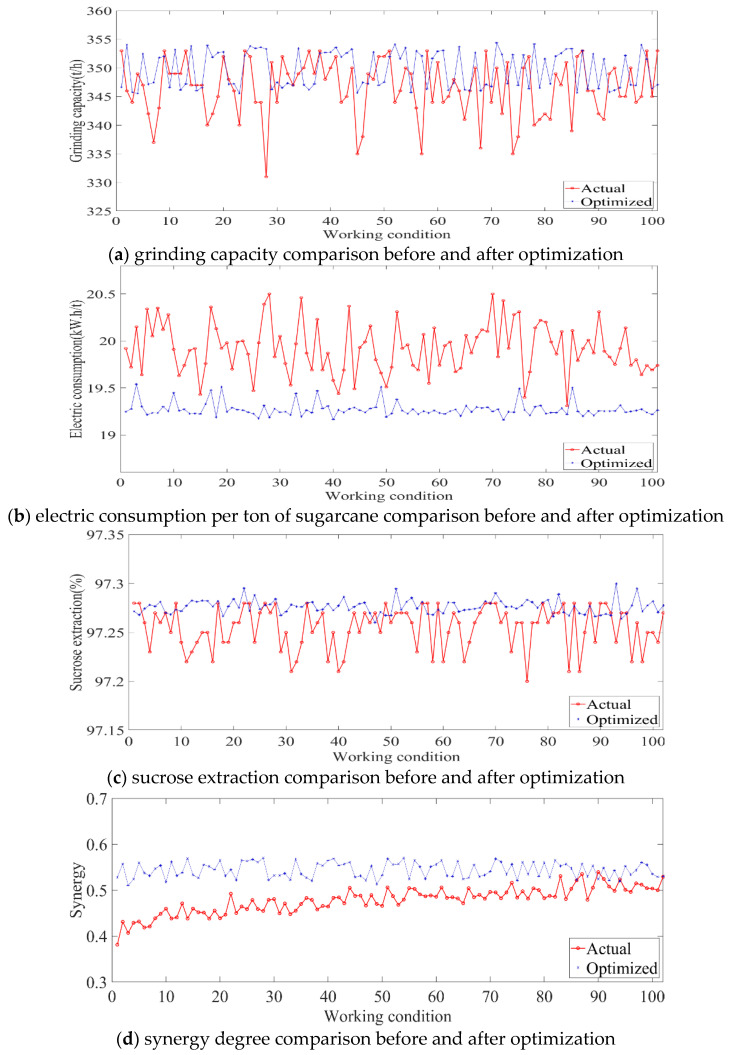
Comparison curves before and after optimization of each control objective for different working conditions in sugarcane milling production process.

The improved DDPG algorithm was used for 102 groups of different working conditions. Figure 18 shows the final optimization results of each operating parameter under different working conditions in the sugarcane milling process. Based on the optimized operating parameter values applied to the corresponding working conditions, the process indices under each working condition were obtained and compared with those before optimization. Figure 19 shows the comparative effects of grinding capacity, electric consumption per ton of sugarcane, sucrose extraction, and synergy degree before and after the optimization of the 102 groups of working conditions.

As shown in Figure 19d, the synergy degree (*SE*) of the selected working conditions before optimization was less than 0.5 under most conditions. After the optimization of the operating parameters, the synergy degree of the working condition index increased. The synergy degree of the selected working conditions was greater than 0.5, and the system gradually moved from disorder to order. As shown in Figure 19a–c, under the different working conditions, the order parameters corresponding to each flow subsystem were greatly optimized after the optimization of the process parameters. The average values of grinding capacity before and after optimization were 346.51 t/h and 349.71 t/h, respectively—An increase of 3.2 t/h. The average values of electric consumption per ton of sugarcane before and after optimization were 19.93 kW.h/t and 19.27 kW.h/t, respectively—A decrease of 660 W.h/t. The average values of the sucrose extraction before and after optimization were 97.25% and 97.28%, respectively—An increase of 0.03%. 

According to expert experience, every 1% increase in sucrose extraction can yield about 1.2 million t more sugar per 100,000 t of cane milled, with a total increase in production value of about $ 120,000. In the last milling season, the sugar mill squeezed about 1.1 million tons of sugarcane, and if the sucrose extraction is estimated to increase by 0.03%, the output value increases by about $ 40,000, with economic significance. In addition, as shown in Figure 18, the optimized setting values for each operating variable met the actual production process requirements. The above results show that this paper’s operating parameter optimization method has a good optimization effect. After the working condition changes, the target value can be tracked in real time, providing a feasible method for the optimization of the operating parameters in the sugarcane milling process.

#### 5.3.2. DDPG Parameter Settings and Optimization Results

**Figure 20:**
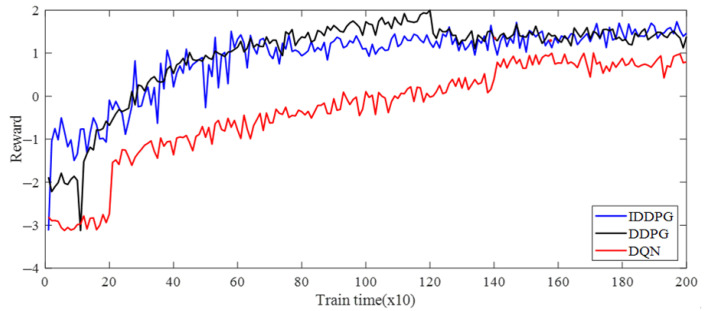
Comparison curves of different algorithm training reward in the sugarcane milling production process.

To validate the effectiveness of the improved DDPG algorithms, the DQN and traditional DDPG algorithms were compared based on the same development framework. To ensure a fair comparison, the same compilation environment was used for all algorithms, and after the agent’s training was completed, the data under the same 102 sets of working conditions were selected to verify the results. The size of the experience pools of both DDPG and DQN was set to 5000, the number of samples selected in the batch was 400, and the discount factor was taken as 0.9. The optimization results of each algorithm are shown in (Table 8), and the reward curve of the training process is shown in Figure 20.

As illustrated in Table 8, the optimization results of 102 sets of order parameters were compared under different working conditions. The optimized grinding capacity and the sucrose extraction of the improved DDPG were improved compared to both conventional DDPG and DQN, while the optimized electric consumption per ton of cane was reduced compared to both conventional DDPG and DQN. Meanwhile, the optimized results obtained by the improved DDPG algorithm were all improved compared to the unoptimized index. As shown in Figure 20, the cumulative reward values of all algorithms fluctuated up and down, but all moved toward a better strategy, with the improved DDPG algorithm having the fastest convergence and the best performance, followed by DDPG, while DQN had the worst effects. The above results show that the improved DDPG can efficiently optimize the operational parameters of the sugarcane milling production process.

## 6. Conclusions

In order to improve the performance of the sugarcane milling system, an optimization framework for sugarcane milling systems was implemented using the combination of the DK-ELM model, a MOCSO algorithm based on flow coordination, and an improved DDPG algorithm. The sugarcane milling system being used in a sugar factory in Guangxi was selected as the research object and abstracted into an MF–EF–IF coordination system. The state representation of the flow subsystems was established based on the workshop operating data. Then, a DK-ELM data-driven model was proposed to effectively regress the relationships between the order parameters and the feature variables of each flow subsystem. A collaborative optimization model was established with the optimization objectives of a high grinding capacity, low electric consumption, and high sucrose extraction, and a collaborative evaluation model based on the MOCSO algorithm was introduced to determine the optimal values of each order parameter. The reward function was established by combining the optimal values of the order parameters and the model of each flow subsystem, and the optimal values of the order parameters were used as the control objectives to optimize the operational parameters of the milling system under different working conditions based on the improved DDPG algorithm. The method’s effectiveness was verified by numerical simulation; the grinding capacity per hour was increased by 3.2 t, the electric consumption per ton of sugarcane was reduced by 660 W, and the sucrose extraction was increased by 0.03%. This provides a new approach for the operational optimization of such complex industrial processes. To improve the accuracy of state representation of each flow, the data-driven model in this paper could be further improved by combining worker experience and mechanism analysis of the production process.

## Figures and Tables

**Table 1:** Operating parameter set of the sugarcane milling system.

Number	Parameter	Description	Number	Parameter	Description
1	*x*_1_	#1Crusher current	15	*x*_15_	#3Squeezer speed
2	*x*_2_	#2Crusher current(East)	16	*x*_16_	#4Squeezer current
3	*x*_3_	#2Crusher current(West)	17	*x*_17_	#4Squeezer speed
4	*x*_4_	#3Crusher current	18	*x*_18_	#5Squeezer current
5	*x*_5_	First-level belt current	19	*x*_19_	#5Squeezer speed
6	*x*_6_	First-level belt speed	20	*x*_20_	#6Squeezer current
7	*x*_7_	Second-level belt current	21	*x*_21_	#6Double roller speed
8	*x*_8_	Second-level belt speed	22	*x*_22_	#6Squeezer speed
9	*x*_9_	Double roller speed	23	*x*_23_	#6Double roller current
10	*x*_10_	#1Squeezer speed	24	*x*_24_	Permeate water flow
11	*x*_11_	#1Squeezer current	25	*x*_25_	Permeate water-to-sugarcane ratio
12	*x*_12_	#2Squeezer current	26	*x*_26_	Sucrose content
13	*x*_13_	#2Squeezer speed	27	*x*_27_	Non-sugar content of cane
14	*x*_14_	#3Squeezer current	28	*x*_28_	Cane fibre

**Table 2:** Optimal parameter results for data-driven models of material, energy and information flow subsystems.

Model Output	Determination Coefficient (*R*^2^)	Model Input	Optimized Parameter Values	
			Penalty Factor (*C*)	Kernel Function Parameter (*γ*)
Grinding capacity	0.9569	{*x*_6_, *x*_8_, *x*_11_, *x*_14_, *x*_20_, *x*_26_, *x*_28_}	[347.0117, 558.520, 24.8699]	[94.3138, 249.1454, 537.7881]
Electric consumption per ton of sugarcane	0.9776	{*x*_4_, *x*_8_, *x*_14_, *x*_15_, *x*_20_, *x*_23_, *x*_25_, *x*_27_}	[187.9431, 1024, 124.5195]	[680.8325, 560.2704, 741.2701]
Sucrose extraction	0.9282	{*x*_4_, *x*_8_, *x*_11_, *x*_14_, *x*_17_, *x*_20_, *x*_21_, *x*_23_, *x*_25_, *x*_27_}	[260.9975, 871.738, 65.3083, 622.3309]	[485.1199, 180.6365, 386.6616, 501.7772]

**Table 3:** Value range of decision variables of the sugarcane squeezing process.

Parameter	Description (Unit)	Min Value	Max Value	Parameter	Description (Unit)	Min Value	Max Value
*x*_4_	#3Crusher current (A)	53	66	*x*_20_	#6Squeezer current (A)	631	804
*x*_6_	First-level belt speed (m/s)	5.5	8.1	*x*_21_	#6Double roller speed (m/s)	3.3	4.2
*x*_8_	Second-level belt speed (m/s)	6.1	7.8	*x*_23_	#6Double roller current (A)	906	1092
*x*_11_	#1Squeezer current (A)	969	1092	*x*_25_	permeate Water-to-sugarcane ratio (null)	15.94	21.72
*x*_14_	#3Squeezer current (A)	788	934	*x*_26_	Sucrose content (%)	14.19	14.91
*x*_15_	#3Squeezer speed (m/s)	4.9	6.2	*x*_27_	Non-sugar content of cane (%)	2.16	2.59
*x*_17_	#4Squeezer speed (m/s)	3.5	5.6	*x*_28_	Cane fibre(%)	10.16	10.5

**Table 4:** Data-driven model performance of the sugarcane milling production process based on DK-ELM.

Model Output	Model Training Time (s)	MAE	RMES	*R* ^2^
Grinding capacity	0.7682	4.9045	5.7333	0.9569
Electric consumption per ton of sugarcane	0.8281	0.1292	0.1646	0.9776
Sucrose extraction	0.9688	0.0271	0.0332	0.9282

**Table 5:** Parameters setting of IMOSCO.

Algorithm Parameters	Values
Size of population	350
Number of iterations	300
Archive capacity	150
Proportion of rooster	0.2
Proportion of hen	0.6
Proportion of chicken	0.2
Learning factor for chick to hen	0.4
Learning factor for chick to rooster	0.6

**Table 6:** Training parameters setting of DDPG.

Training Parameters	Values
Size of experience pool	5000
Number of cycles in the outer layer	2000
Time step of inner layer	7
Adjustment time of inner action space	60
Threshold of discarding the sample	0.5
Discount factor	0.9
Learning rates of actual network	0.0001
Learning rates of target network	0.01
Soft update rate	0.001
Exploration noise	0.01

**Table 7:** Neural network parameters setting of DDPG.

Network Name	Network Layers	Activation Function	Number of Neurons
policy network	2	ReLU, tanh	80,30
Q network	2	ReLU, tanh	80,30
target policy network	2	ReLU, tanh	100,35
target Q network	2	ReLU, tanh	100,35

**Table 8:** Optimal results of each algorithm.

Order Parameter	Actual Average Value	DQN	DDPG	Improved DDPG
Grinding capacity	346.51	338.08	347.87	349.71
Electric consumption per ton of sugarcane	19.93	20.11	19.29	19.27
Sucrose extraction	97.25	97.20	97.26	97.28

## Data Availability

Data available on request due to restrictions eg privacy or ethical. The data presented in this study are available on request from the corresponding author. The data are not publicly available due to confidentiality agreement was signed because the data is an important confidential document for the sugar factory‘s processing and production.
